# Predictors of Attrition in Patients Ineligible for Antiretroviral Therapy after Being Diagnosed with HIV: Data from an HIV Cohort Study in India

**DOI:** 10.1155/2013/858023

**Published:** 2013-08-29

**Authors:** Gerardo Alvarez-Uria, Manoranjan Midde, Raghavakalyan Pakam, Praveen Kumar Naik

**Affiliations:** Department of Infectious Diseases, RDT Bathalapalli Hospital, Kadiri Road, Bathalapalli 515661, India

## Abstract

In newly HIV-diagnosed patients, the CD4+ lymphocyte count is measured to determine the need for antiretroviral therapy (ART). Studies from Sub-Saharan Africa have shown that patients who are ART ineligible at the first assessment have poor retention in care, but data from other low- or middle-income countries are scarce. In this study we describe the retention in pre-ART care of 1696 patients who were ineligible for ART after being diagnosed with HIV in a cohort study in India. More than one-third of ART ineligible patients had poor retention in care, and the attrition was higher in those with longer follow-up periods. Of those patients with poor retention, only 10% came back to the clinics, and their CD4 cell counts were lower than the ones of patients retained in care. After 4.5 years of follow-up, the cumulative incidence of loss to follow-up was 50%. Factors associated with attrition were being homeless, being illiterate, belonging to a disadvantaged community, being symptomatic at the time of the HIV diagnosis, male gender, and not living near a town. Widows were given nutritional support and, therefore, had better retention in care. The results of this study highlight the need to improve the retention in care of ART ineligible patients in India.

## 1. Introduction

More than 90% of the 34 million people infected with HIV live in low- or middle-income countries [1]. With the roll-out of antiretroviral therapy (ART), most HIV-infected people living in these countries have free access to HIV treatment. Nevertheless, 1.7 million people died of HIV-related pathologies in 2011 [1]. 

After HIV diagnosis, a CD4+ lymphocyte count measurement is done to determine whether patients need to start ART or not, and a substantial proportion of patients are ineligible for ART initiation [2]. These patients are followed up until they become eligible for ART, in order to initiate ART before the development of HIV-related pathologies. Previous studies from Sub-Saharan Africa have shown that ART ineligible patients have poor retention in pre-ART care [3–5], and many of them are lost to follow-up or may reengage in care again only after developing severe opportunistic infections [6–8]. 

With 2.4 million HIV-infected people [9], India has the third largest burden of HIV worldwide, but data of the retention in care of patients ineligible for ART at the first assessment are lacking. This study describes the retention in care of these patients in a large cohort study in Anantapur, India.

## 2. Methods

### 2.1. Setting

The study was performed in Anantapur, a district situated in the State of Andhra Pradesh, India. Anantapur has a population of approximately four million people, and 72% of them live in rural areas [10]. Rural Development Trust (RDT) is a nongovernmental organization that provides medical care to HIV-infected people free of cost, including medicines, consultations, or hospital admission charges. RDT has three hospitals in the district, and patients diagnosed with HIV are referred to Bathalapalli RDT Hospital, where CD4 cell count enumeration and ART are provided free of cost by the Indian Government under a public-private partnership. During the study period, ART was also provided by another ART centre in the district (Anantapur ART centre). The Vicente Ferrer HIV Cohort Study (VFHCS) is an open cohort study of all HIV-infected patients who have attended RDT hospitals since June 2006. The characteristics of the cohort have been described in detail elsewhere [11]. The cohort is fairly representative of the HIV population in the district, because it covers approximately 70% of all HIV-infected patients registered in the district [12].

### 2.2. Design

For this study, we selected HIV-infected adults (>15 years) found to be ineligible for ART at the first assessment between January 1st, 2007, and March 25th, 2011. During this period, ART was available free of cost in the district, and the CD4 cell count threshold for initiating ART was 250 cells/*μ*L [13–15]. CD4 cell count determinations were performed on a daily basis. Patients were requested to come back to the clinics after one or two days or to wait until evening to collect the results of the CD4 cell count.

To assess the retention in pre-ART care we followed recent recommendations based on the experience of studies performed in Sub-Saharan Africa [6, 16]. For HIV-infected patients who are ineligible for ART at the first assessment, the Indian National Guidelines recommend performing CD4 cell count determinations every six months [13]. To describe the retention in pre-ART care of these patients, we calculated the number of completed semesters in which patients had a CD4 cell count determination during the period starting at three months after the first CD4 cell count determination and finishing at the moment of becoming eligible for ART (CD4 cell count < 250 cells/*μ*L), death, or November 4th, 2011, whatever occurred first (follow-up period). To be considered retained in pre-ART care, patients should have had a CD4 cell count determination in all the semesters of follow-up. However, patients having a CD4 cell count > 250 cells/*μ*L in the last 12 months of follow-up were considered retained in pre-ART care regardless of the number of previous semesters without CD4 cell count determinations [16]. Patients should have had a follow-up period of at least six months to be included in the study. For example, in a patient who had the first CD4 cell count determination on October 1st, 2009, and died on January 15th, 2011, we checked whether the patient had a CD4 cell count determination from January 1st, 2010 (three months after the first CD4 cell count determination) to June 30th, 2010 (first semester) and from July 1st, 2010, to December 31st, 2010 (second semester). If he had CD4 cell count determinations during the first and second semesters, the patient was considered retained in care. If he had a CD4 cell count determination only in one semester, he did not meet criteria for being considered retained in pre-ART care. However, if the same patient had a CD4 lymphocyte count > 250 cells/*μ*L in the last 12 months of follow-up, he was considered retained in care, even if he did not have CD4 cell count determinations in all the semesters of follow-up.

### 2.3. Definitions

Designation of the community of patients was performed by self-identification. Scheduled caste community is marginalised in the traditional Hindu caste hierarchy and, therefore, suffers social and economic exclusion and disadvantage. Scheduled tribe community is generally geographically isolated with limited economic and social contact with the rest of the population. Scheduled castes and scheduled tribes are considered socially disadvantaged communities and are supported by positive discrimination schemes operated by the Government of India [17]. Patients were considered as living near an ART centre if they lived in a mandal (administrative subdivision of districts in India; e.g., Anantapur district has 64 mandals) with an ART centre or lived next to a mandal with an ART centre. ART centres are healthcare facilities able to perform CD4 cell count and provide ART to patients. During the study period, there were two mandals with an ART centre in the district. Patients were considered as living near a town when they lived in one of the six mandals containing a town with a population >100,000. Towns have better communications than rural areas. Poverty was defined as living with less than 1000 Indian rupees per month (approximately 18 US dollars in April 2013). Patients who had poor retention in care and who did not come back to the clinics during the study period were considered as lost to follow-up.

### 2.4. Statistical Analysis

Statistical analysis was performed using Stata Statistical Software (Stata Corporation, Release 11, College Station, TX, USA). To include all cases in the multivariable analysis, missing values were imputed using multiple imputations by chained equation assuming missing at random [18]. Adjusted risk ratios were calculated using Poisson regression with robust variance [19]. While performing Poisson regression, we found interactions between gender and other variables. Hence, stratified-by-gender analysis was also performed. The cumulative incidence of loss to follow-up and ART eligibility was estimated using competing-risk analysis (stcompet command in Stata) [20, 21]. The study was approved by the Ethical Committee of the RDT Hospital. 

## 3. Results

We identified 1696 patients ineligible for ART at the first assessment who met the inclusion criteria for the study. 446 (26.3%) had CD4 cell count measurements in all semesters of follow-up. Of 1250 patients who did not have CD4 cell count measurements in all semesters, 662 (53%) had a CD4 cell count > 250 cells/*μ*L in the last 12 months of follow-up. Hence, 1108 (65.3%, 95% CI 63–67.3) were considered as retained in pre-ART care according to study criteria. The median follow-up period since the first CD4 cell count determination was 25.6 months (interquartile range (IQR), 16.2–37.4). During the study period, 579 patients became eligible for ART, 13 died, and 1104 were censored on November 4th, 2011. 531 (90%) patients with poor retention were lost to follow-up, and 57 (10%) came back to the clinics when their CD4 cell count was <250 cells/*μ*L. The CD4 cell count at ART eligibility of patients with poor retention (median 177 cells/*μ*L, IQR 119–210) was significantly lower (Wilcoxon rank sum test *P* = 0.018) than that of patients retained in care (median 194 cells/*μ*L, IQR 148–219).

Baseline characteristics and factors associated with retention in pre-ART care overall and by gender are presented in Table 1. Over half of patients were women, and two-thirds were <35 years old. Over one-quarter belonged to disadvantaged communities, more than half were illiterate, and over two-thirds were married. Eight percent were homeless, over one-third were living near an ART centre, 46% were living near a town, and 42% were poor. In 42% of the cases the follow-up period was <1 year, and three-quarters had a CD4 cell count > 350 cells/*μ*L at the first assessment. Near half of patients presented symptoms at the moment of HIV diagnosis.

Factors associated with retention in pre-ART care were being widowed, poverty (risk ratio (RR) 1.08, 95% CI 1.01–1.16), female gender (RR 1.09, 95% CI 1–1.18), and living near a town (RR 1.09, 95% CI 1.01–1.16). Factors associated with poor retention in pre-ART care were longer duration of follow-up, being homeless (RR 0.56, 95% CI 0.45–0.69), being illiterate (RR 0.88, 95% CI 0.83–0.95), and being symptomatic at the moment of HIV diagnosis (RR 0.9, 95% CI 0.84–0.97). Belonging to a disadvantaged community was associated with poor retention only in women (RR 0.84,95% CI 0.76–0.94). 

**Figure 1:**
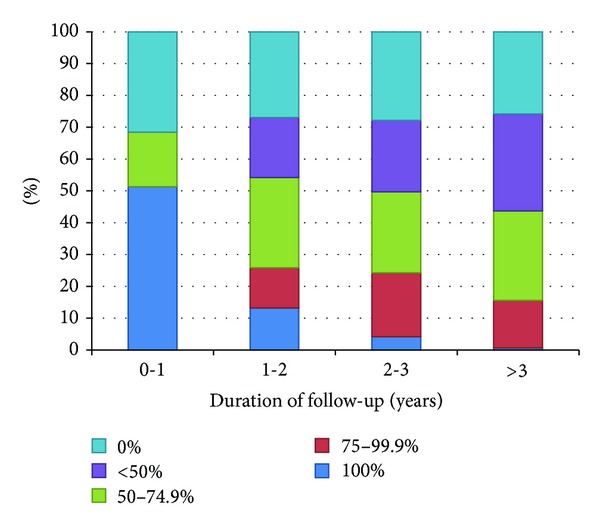
Proportion of semesters with CD4 cell count determinations of patients ineligible for antiretroviral therapy by duration of follow-up. Patients were divided into four groups according to their follow-up period: less than one year, one to two years, two to three years, and more than three years. The percentages indicate the proportion of semesters in which these patients had a CD4 cell count determination.

The proportion of semesters with a CD4 cell count determination by duration of follow-up is presented in Figure 1. While the proportion of patients who did not have any CD4 cell count determination was slightly reduced over time, the proportion of patients who had a CD4 cell count determination in more than half of the semesters was reduced in patients with longer follow-up periods.

**Figure 2:**
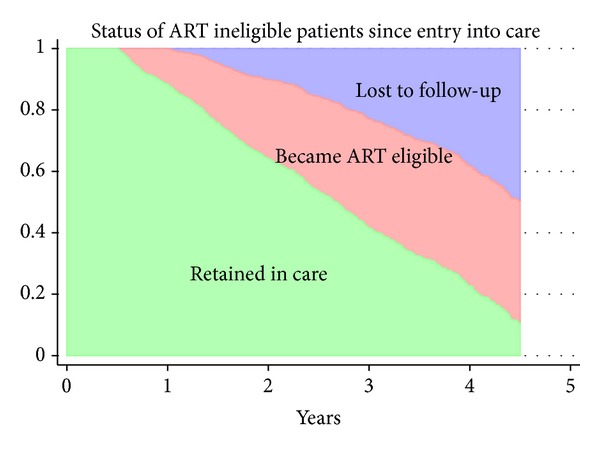
Cumulative incidence of loss to follow-up and antiretroviral therapy eligibility of patients ineligible for antiretroviral therapy at the first assessment. ART: antiretroviral therapy.

Figure 2 shows the cumulative incidence of ART eligibility and loss to follow-up over time. It was estimated that after 4.5 years of follow-up, 39.8% (95% CI, 37.1–42.4) became eligible for ART and 50% (95% CI, 47.7–53.2) were lost to follow-up.

## 4. Discussion 

In this study, more than one-third of HIV-infected patients who entered into care and were ineligible for ART had poor retention in care, and, after 4.5 years of follow-up, it was estimated that 50% were lost to follow-up. These findings are in line with a recent meta-analysis from Sub-Saharan Africa, where the estimated proportion of loss to follow-up among ART ineligible patients was 57.3% (95% CI, 34.3–80.2) [22]. These results indicate that there is an urgent need to improve the retention in pre-ART care of ART ineligible patients in India and other resource-limited settings. 

This is one of the first studies to describe risk factors of poor retention in care among ART ineligible patients in India. Living far from urban areas, longer follow-up periods, and variables associated with poor socioeconomical status (being homeless, being illiterate and belonging to a disadvantaged community) were the most important factors associated with pre-ART attrition. 

Symptomatic patients at HIV diagnosis were more likely to have poor retention in pre-ART care if they were not given ART after the first assessment in the clinics. These patients might have sought a treatment for their symptoms in other health-care facilities and, therefore, were less likely to return to the clinics. 

Widows and poor women were more likely to remain in pre-ART care, but the site offers nutritional support to these patients, so these findings cannot be generalized to other sites. In Kenya, the free provision of cotrimoxazole among ART ineligible patients improved the 12-month retention in care from 63% to 84%, suggesting that these patients may have perceived more benefit from clinic attendance because of the cotrimoxazole treatment [23]. These data indicate that the provision of free medicines or nutrition can improve the usefulness perception of the visits to the clinic and, therefore, the retention in pre-ART care.

In contrast to two South-African studies with shorter follow-up periods [3, 4], in this study, younger age and higher initial CD4 cell counts were not associated with attrition, but our study agrees with these South-African studies in that men were more likely to have poor retention. Although women were more likely to be retained in care, belonging to a disadvantaged community and living far from a town were more important factors for women than for men. These data add more evidence about the important gender differences seen in the HIV epidemic in India [11, 24].

The study has some limitations. Patients lost to follow-up may not be lost forever, as they may reengage in the future or enroll in other HIV centres. However, in this study, patients with poor retention who came back to the clinics and were found to be eligible for ART had lower CD4 cell counts than patients who were retained in care, and previous studies have demonstrated a high mortality in patients lost to follow-up [8]. 

## 5. Conclusions

This study highlights the need to improve the retention in care of HIV-infected patients ineligible for ART at the first assessment. After 4.5 years of follow-up, 50% of these patients are lost to follow-up. Factors associated with poor retention were being homeless, being illiterate, belonging to a disadvantaged community, being symptomatic, male gender, and living in rural areas. These findings can be used to improve the retention in pre-ART care in India, by giving more support to those patients at higher risk of attrition.

## Figures and Tables

**Table 1:** Baseline characteristics and factors associated with retention in preantiretroviral therapy care.

	Baseline characteristics	Retention in care	Multivariable analysis of factors associated with retention in pre-ART care		
	(*n* = 1696)	(65.3% overall)	Overall	Males	Females
	*N* (%)	*N* (%)	aRR (95% CI)	aRR (95% CI)	aRR (95% CI)
Age (years)					
<25	471 (27.77)	294 (62.42)	0.93 (0.85–1.01)	0.93 (0.76–1.14)	0.94 (0.86–1.03)
25–35	795 (46.88)	537 (67.55)	1 (reference)	1 (reference)	1 (reference)
35–45	312 (18.4)	205 (65.71)	1.00 (0.91–1.09)	0.97 (0.85–1.11)	1.03 (0.92–1.16)
>45	118 (6.96)	72 (61.02)	0.95 (0.82–1.10)	0.96 (0.80–1.15)	0.92 (0.72–1.18)
Disadvantaged community	447 (26.36)	272 (60.85)	0.91* (0.84–0.99)	1.02 (0.90–1.16)	0.84* (0.76–0.94)
Illiteracy	963 (56.88)	607 (63.03)	0.88* (0.83–0.95)	0.85* (0.76–0.95)	0.91* (0.84–0.99)
Marital status					
Divorced/separated	76 (4.49)	44 (57.89)	0.94 (0.77–1.15)	0.90 (0.65–1.25)	0.97 (0.76–1.23)
Married	1206 (71.23)	777 (64.43)	1 (reference)	1 (reference)	1 (reference)
Unmarried	103 (6.08)	50 (48.54)	0.83 (0.68–1.02)	0.83 (0.67–1.03)	1.07 (0.65–1.77)
Widowed	308 (18.19)	235 (76.3)	1.18* (1.09–1.28)	1.22 (0.99–1.50)	1.17* (1.08–1.28)
Homeless	127 (7.78)	48 (37.8)	0.56* (0.45–0.69)	0.53* (0.37–0.75)	0.58* (0.44–0.76)
Living near an ART centre	602 (35.5)	400 (66.45)	1.03 (0.96–1.10)	1.03 (0.92–1.16)	1.03 (0.95–1.13)
Living near a town	785 (46.29)	535 (68.15)	1.09* (1.01–1.16)	1.06 (0.94–1.18)	1.11* (1.02–1.20)
Poverty	691 (41.98)	469 (67.87)	1.08* (1.01–1.16)	1.06 (0.94–1.19)	1.10* (1.01–1.20)
Follow-up period (years)					
0-1	719 (42.39)	537 (74.69)	1 (reference)	1 (reference)	1 (reference)
1-2	472 (27.83)	290 (61.44)	0.81* (0.74–0.88)	0.76* (0.66–0.87)	0.85* (0.76–0.94)
2-3	338 (19.93)	191 (56.51)	0.75* (0.68–0.83)	0.66* (0.56–0.79)	0.82* (0.73–0.93)
>3	167 (9.85)	90 (53.89)	0.70* (0.61–0.80)	0.51* (0.37–0.70)	0.82* (0.71–0.95)
First CD4 count					
<350	426 (25.12)	267 (62.68)	0.92 (0.84–1.01)	0.87 (0.75–1.01)	0.97 (0.87–1.09)
350–500	619 (36.5)	414 (66.88)	1.00 (0.93–1.08)	0.98 (0.86–1.11)	1.03 (0.94–1.13)
>500	651 (38.38)	427 (65.59)	1 (reference)	1 (reference)	1 (reference)
Symptomatic at HIV diagnosis	808 (47.64)	481 (59.53)	0.90* (0.84–0.97)	0.93 (0.83–1.04)	0.90* (0.82–1.00)
Female gender	923 (54.42)	644 (69.77)	1.09* (1.00–1.18)		

*P value <0.05. ART: antiretroviral therapy; aRR: adjusted risk ratio; CI: confidence interval.
